# Adherence to Post-Exposure Prophylaxis (PEP) and Incidence of HIV Seroconversion in a Major North American Cohort

**DOI:** 10.1371/journal.pone.0142534

**Published:** 2015-11-11

**Authors:** Réjean Thomas, Chrissi Galanakis, Sylvie Vézina, Danièle Longpré, Michel Boissonnault, Emmanuelle Huchet, Louise Charest, Daniel Murphy, Benoît Trottier, Nimâ Machouf

**Affiliations:** Clinique médicale l’Actuel, Montreal, Quebec, Canada; University of Pittsburgh Center for Vaccine Research, UNITED STATES

## Abstract

**Background:**

There is limited evidence on the efficacy of post-exposure prophylaxis (PEP) for sexual exposures. We sought to determine the factors associated with adherence to treatment and describe the incidence of PEP failures in a Montreal clinic.

**Methods:**

We prospectively assessed all patients consulting for PEP following sexual exposures from October 2000 to July 2014. Patients were followed at 4 and 16 weeks after starting PEP. Treatment adherence was determined by self-report at week 4. Multivariable logistic regression was used to estimate the factors predicting adherence to treatment.

**Results:**

3547 PEP consults were included. Patients were mainly male (92%), MSM (83%) and sought PEP for anal intercourse (72%). Seventy-eight percent (n = 2772) of patients received a prescription for PEP, consisting of Tenofovir/Emtracitabine (TVD) + Lopinavir/Ritonavir (LPV) in 74% of cases, followed by Zidovudine/Lamivudine (CBV) + LPV (10%) and TVD + Raltegravir (RAL) (8%). Seventy percent of patients were adherent to treatment. Compared to TVD+LPV, patients taking CBV+LPV were less likely to adhere to treatment (OR 0.58, 95% CI 0.44–0.75), while no difference was observed for patients taking TVD+RAL (OR 1.15, 95% CI 0.83–1.59). First-time PEP consults, older and male patients were also more adherent to treatment. Ten treated patients seroconverted (0.37%) during the study period, yet only 1 case can be attributed to PEP failure (failure rate = 0.04%).

**Conclusion:**

PEP regimen was associated with treatment adherence. Patients were more likely to be adherent to TVD-based regimens. Ten patients seroconverted after taking PEP; however, only 1 case was a PEP failure as the remaining patients continued to engage in high-risk behavior during follow-up. One month PEP is an effective preventive measure to avoid HIV infection.

## Introduction

The estimated risk of HIV transmission via sexual intercourse ranges from 0.2 to 1.4% depending on the type of exposure, with receptive anal intercourse having the greatest risk of HIV acquisition [1–4]. The indication of Post-Exposure Prophylaxis (PEP) following sexual exposures has been recommended by various international guidelines though current evidence on PEP efficacy regarding these exposures is sparse and most research supporting its use stems from animals models and observational studies on mother-to-child transmission and occupational exposures [3, 5–8].

Adherence and continued high-risk sexual behaviors are both key determinants of PEP efficacy [3]. However, treatment completion rates are inconsistent across the literature and vary according to exposure, population and study type [9–15]. Moreover, though studies have described the incidence of HIV infection following PEP use for non-occupational exposures, it is unclear whether the seroconversions that occurred are the result of PEP failure or continued exposure to HIV [16, 17]. While a 28-day regimen of PEP administered within 72 hours of the exposure may prevent HIV infection, its effectiveness for sexual exposures remains unknown [6, 18, 19]. As poor adherence and re-exposure to HIV limit the assessment of PEP efficacy, determining the factors associated with the latter is crucial.

In this article we aimed to: 1) describe the population presenting for PEP following sexual exposures at our clinic; 2) determine the factors associated with adherence to PEP treatment and 3) document the incidence of the seroconversions from treatment failures.

## Methods

### Study Design and Participants

Clinique médicale L’Actuel is a community clinic specialized in HIV that has been providing PEP for non-occupational exposures since 2000 and receives 40–50 consultations each month. It has one of the largest cohorts of PEP users in Canada with over 4000 consultations to date. We conducted a prospective observational study of all patients presenting for PEP following a sexual exposure from October 2000 to July 2014. We prospectively assessed all patients seeking PEP at their initial visit. Patients refusing consent and those presenting for non-sexual exposures were excluded from the study.

### Clinical Protocol

#### Initial Patient Evaluation

Upon presentation at the clinic, information regarding the sexual exposure, consultation delay, the source and the patient’s history of at-risk behavior was evaluated by a triage nurse. Patients were also explained the follow-up procedure and provided counseling on antiretroviral (ARV) therapy. HIV rapid testing (INSTI), MEIA screening for HIV antibodies and antigen p24 tests (Abbott ARCHITECT ® HIV Ag/Ab Combo) were offered to patients and their partners, if present at the consultation. Patients were then examined by a physician, attributed an exposure risk (negligible, moderate or high) and indicated a treatment accordingly. Advice on ARV use and STD counseling was also provided to patients at this time.

#### Treatment

Patients were prescribed a 28-day ARV regimen consisting mainly of 2 NRTIs and a third agent (protease inhibitor or integrase inhibitor). Treatment was not indicated for patients who presented 72 hours or more after the exposure, for those with a negligible risk of infection or for those testing HIV positive at baseline.

#### Follow-up and Adherence

Patients receiving treatment had scheduled follow-up visits at 4 weeks (end of treatment) and 12 weeks after the initial PEP consultation. Prior to 2009, the PEP protocol consisted of 8 visits over a 6-month follow-up period (24 weeks). From 2009 to February 2014, the PEP follow-up changed to 3 visits over a 4-month period (16 weeks). Thus, for the purpose of this study, we used the information collected at baseline, week 4 and either week 12, 16 or 24 depending on the year of the PEP consult.

Information on treatment adherence, side-effects and ongoing at-risk sexual behavior was collected and HIV testing was performed at each follow-up visit. Patients were considered adherent to treatment if they did not miss more than 5 doses during their month-long treatment. Ongoing at-risk sexual practices were defined as unprotected anal sexual intercourse, fellatio with receptive ejaculation or any other behavior the physician evaluated to be high risk.

### Sexual Exposure

We collected information on the type of intercourse (e.g. anal, vaginal, fellatio, with or without ejaculation, receptive or insertive), condom use, drug and alcohol use at the time of exposure, relationship with the source, HIV status of the source and the risk status of the source. HIV status was defined as positive, negative (if the source was tested at the visit), or unknown with either low or high risk of being HIV positive. A source was considered high risk if they belonged to the following groups: men who have sex with men (MSM), sex workers, from endemic regions, hemophiliacs or intravenous drug users.

### Ethical Considerations

Ethical approval was provided by the Veritas Independent Review Board. Patients provided written consent to the use of their clinical data for research purposes on post-exposure prophylaxis.

### Statistical Analysis

Statistical analysis was performed using SPSS version 17.0 (IBM, USA). Multivariable logistic regression was used to estimate the factors predicting adherence to treatment. Intent-to-treat analysis was applied to treatment adherence. P-values less than 0.05 were considered statistically significant.

## Results

As of July 1^st^ 2014, we received 3700 PEP consultations. Among the consultations, 153 were excluded: 74 patients refused consent, 7 patients left the consultation before they could be evaluated by a physician, and 72 consultations were for non-sexual exposures. A total of 3547 consultations were included in this study.

Patients consulting for sexual PEP were primarily male (92%), MSM (83%) and university educated (49%) with a mean age of 34.6 (SD 10.2). It was the first consultation for 70% of patients, 25% had 2–4 PEP consultations and only 3% of patients had 5 or more PEP episodes. Forty-three percent of patients were intoxicated at the time of exposure and two-thirds of patients had sexual relations with an unknown source (Table 1). Among exposures where the patient was familiar with the source, the source was known to be HIV+ in 64% of cases, whereas 15% tested HIV-negative and 21% had unknown HIV statuses. The risk of the exposure was considered moderate to high in 81% of cases. Anal intercourse was the most common type of exposure (n = 2560, 72%), followed by fellatio (n = 1607, 45%), receiving fellatio (n = 1188, 33%) and vaginal intercourse (n = 542, 15%). Over half (54%) of anal intercourse was receptive, 56% of which was unprotected. Likewise, 51% of insertive anal intercourse and 43% of vaginal intercourse was unprotected. All except 51 (1%) patients consulted within the 72-hour delay following the exposure.

**Table 1:** Patient and exposure characteristics (N = 3547).

Characteristic	N	%
**Age (mean, Range)**	34.6	(18–76)
**Male**	3245	92
**MSM**	2933	83
**Education level**		
High school or less	535	15
College	776	22
University	1730	49
Missing	507	14
**Consultation delay (hours)**		
< 24	1719	48
25–48	1189	34
49–72	558	16
> 72	51	1
Missing	30	1
**No. PEP episodes**		
First episode	2497	70
2–4 episodes	881	25
≥ 5 episode	107	3
Missing	62	2
**Intoxicated during intercourse**	1530	43
**Risk of exposure**		
Low	647	18
Moderate to High	2883	81
Missing	17	1
**Source known to patient**	1184	33
**HIV + source** (confirmed)*	753	64
**Serodiscordant couple**	132	4
**Violence aggression**	101	3

* Among sources which are known to patients (n = 1184).

Among the 3547 consults, 2772 (78%) patients received a prescription for PEP (Table 2). Patients were mainly prescribed 2 NRTIs and a protease inhibitor, consisting of Tenofovir/Emtacitabine (TVD) + Lopinavir/Ritonavir (LPV) (74%) or Zidovudine/Lamivudine (CBV) + LPV (10%). TVD + Raletegravir (RAL) was prescribed in 8% of cases. The exposure risk was perceived as moderate to high in 96% of cases where treatment was indicated (data not shown). However, only 2731 patients were treated as 41 stopped their treatment prematurely as the source tested HIV negative and no window period was suspected. Of the 2731 treated patients, 69% completed their entire prescribed treatment, 2% missed more than 5 doses, 4% discontinued prophylaxis and 1% switched to a different regimen, while 16% were lost to follow-up and 8% has missing information. Side effects were the main reason for discontinuation and regimen switching (70% and 90% respectively).

**Table 2:** PEP regimen prescribed (N = 2772).

Treatment Regimen	N	%
**TVD + LPV**	2062	74
**CBV + LPV**	275	10
**TVD + RAL**	217	8
**Other combinations**	206	7
**Missing**	12	<1

TVD: Truvada (tenofovir-emtricitabine), CBV: Combivir (zidovudine-lamivudine), LPV: lopinavir/ritonavir, RAL: raltegravir.

Overall, 1902 (70%, OT = 87%) patients were adherent to treatment (Table 3). Patients taking TVD-based regimens adhered more to treatment than those on CBV-based or other regimens (72% adherent vs. 60% and 59%, p <0.001). Compared to patients who received TVD+LPV, patients taking CBV+LPV were less likely to adhere to treatment (OR 0.58, 95% CI 0.44–0.76). Patients taking TVD+RAL, however, were as likely as those taking TVD+LPV to be adherent to treatment (OR 1.15, 95% CI 0.83–1.59). First-time PEP consults, older and male patients also tend to adhere more to treatment (Table 4).

**Table 3:** Adherence to prescribed PEP regimen (N = 2731).

Treatment Regimen	Adherent		Non-Adherent		
	N	%	N	%	
**CBV-based regimens** ^1^	233	60	158	40	p <0.001
**TVD-based regimens** ^2^	1650	72	654	28	
**Other**	17	59	12	41	
**Total**	**1900**	**70**	**824**	**30**	

^1^CBV-based regimens include CBV in combination with lopinavir, nelfinavir, NNRTIs or other protease and integrase inhibitors.

^2^TVD-based regimens include TVD in combination with lopinavir, raltegravir, NNRTIs or other protease and integrase inhibitors.

Information regarding prophylaxis regimen was missing for 7 cases.

**Table 4:** Factors associated with adherence to PEP regimen (N = 2731).

	OR*	95% CI	p	AOR**	95% CI	p
**TVD + LPV**	*Ref*	—-	—-	*Ref*	—-	—-
**CBV + LPV**	0.59	0.46–0.77	<0.001	0.58	0.44–0.75	<0.001
**TVD + RAL**	1.12	0.81–1.54	0.505	1.15	0.83–1.59	0.406
**Other regimen combinations**	0.61	0.46–0.83	0.001	0.66	0.48–0.89	0.007
**Male**	1.98	1.50–2.62	<0.001	1.94	1.46–2.59	<0.001
**Age (per additional year)**	1.02	1.01–1.03	<0.001	1.02	1.01–1.03	<0.001
**1** ^**st**^ **PEP consult**	1.15	0.96–1.37	0.128	1.31	1.09–1.57	0.004
**Moderate/high exposure risk**	1.20	0.81–1.80	0.365	1.05	0.69–1.60	0.799

*Univariate odds ratio

**Adjusted odds ratio

Multivariable logistic regression model of adherence to PEP regimen.

### Seroconversion

Eleven patients seroconverted within the follow-up period of the PEP protocol. One patient, however, was not treated as the source was presumed to be HIV-negative. As such, 10 of the 2731 treated patients could be possible treatment failures (0.37%). Nevertheless, 9 (90%) of the 10 treated cases continued to exhibit high-risk behavior following treatment; therefore, only one case can be considered a pure treatment failure (0.04%). Characteristics of these cases are presented in Table 5. Seroconverted patients had a mean age of 31 years (SD 9.1) and were all male and MSM. It was the first PEP episode for 9 (82%) patients and 6 (55%) consulted within the first 24 hours of the exposure. The main indication for PEP was unprotected anal intercourse (82% receptive, 27% insertive). One patient had no recollection of the exposure and eight (73%) patients were intoxicated at the time of exposure. All treated patients completed treatment; 9 (90%) were compliant and one case has no information on treatment adherence.

**Table 5:** Characteristics of seroconverted cases (N = 11).

Age	Year	Delay (hrs)	Exposure context	Status of Source	Treated	Compliant	Re-exposure	W0	W4	W12	W16	W24
40	2005	—-	URAI	Presumed HIV-	No	NA	NA	neg	—-	**POS**		
41	2007	44	URAI	Unknown	Yes	Yes	Yes	neg	neg	**POS**		
40	2005	54	URAI	Unknown	Yes	Yes	Yes	neg	neg	neg	—-	**POS**
21	2005	5	URAI	HIV+, untreated	Yes	Yes	Yes	neg	neg	—-	—-	**POS**
36	2010	19	UIAI	Unknown	Yes	Yes	Yes	neg	neg	**POS**		
41	2007	37	UIAI	HIV+, untreated	Yes	No	Yes	neg	—-	—-	—-	**POS**
30	2007	9	URAI	Unknown	Yes	Yes	Yes	neg	neg	neg	—-	**POS**
27	2011	21	URAI with multiple sources	Unknown	Yes	Yes	Yes	neg	neg	**POS**		
40	2009	51	URAI	HIV+, treatment unknown	Yes	Yes	**NO**	neg	neg	**POS**	—-	
18	2011	52	URAI	Unknown	Yes	Yes	Yes	neg	neg	**POS**		
33	2014	20	UIAI	Unknown	Yes	—-	Yes	neg	—-	—-	**POS**	

Characteristics of cases with documented seroconversion. Delay denotes consultation delay following the sexual exposure. URAI: Unprotected receptive anal intercourse. UIAU: Unprotected insertive anal intercourse. W0-W24: HIV test results from week 0 to week 24. Neg: HIV-negative test result. POS: HIV-positive test result. Missing information was left blank.

## Discussion

Patients presenting at our clinic are young MSM seeking PEP following high-risk sexual exposures. Unprotected anal intercourse with a source of unknown HIV status was the most common type of exposure among PEP consults. Over half of patients did not use condoms during anal intercourse; a trend that has been reported in other studies [20, 21]. While PEP awareness and utilization have been described as low [22–26], patients in this study seem to be knowledgeable with regards to HIV prophylaxis as almost half consulted within 24 hours of the exposure.

Adherence in this study (70%) was consistent with other PEP completion rates reported in the literature [9, 10, 12, 15, 21, 26, 27]. Treatment regimen was significantly associated with PEP adherence. Among the 3 main regimens prescribed, patients taking CBV+LPV were less likely to adhere to treatment as compared to TVD+LPV regimens and there was no difference in adherence between TVD+LPV and TVD+RAL. Our results indicate that tolerability may be an important factor in improving adherence to PEP. For instance, the observed increase in PEP completion with TVD-based regimens is likely due to the better tolerability of the TDF/FTC backbone [28–31]. Moreover, studies examining PEP regimens consisting of TVD + RAL have also demonstrated fewer side effects among patients taking CBV-based regimens [32] and showed no difference in adherence when compared to TVD alone [13]. Though it is also suggested that 2-drug regimens are associated with improved PEP adherence in terms of pill burden and dosing [3, 9, 21], the latter do not seem to be factors in our patient population as very few patients were prescribed dual therapy. Additional studies are required to assess the effects of pill burden and tolerability of new PEP regimen combinations on treatment adherence.

Moreover, less than 1% of patients seen over the course of 14 years seroconverted following PEP. Though 10 treated patients seroconverted during the study period, we can only attribute one of these cases to possible treatment failure as only one patient reported no additional high-risk exposures. Nevertheless, the possibility remains that other exposures prior to PEP consultation may have resulted in seroconversion. The rate of seroconversion in this study was comparable to other studies on PEP efficacy [12, 17, 19, 26, 33–35]. As in our study, only a small portion of seroconversions were considered PEP failures as the patients continued to exhibit ongoing high-risk sexual behaviors [12, 17, 19, 34]. A longitudinal study of HIV infection after PEP use had 39 (4.4%) cases seroconvert (HIV incidence rate 2.2 infections per 100 person-years) over a 16 year period with the majority of cases seroconverting outside the standard PEP follow-up period [36]. These findings suggest that the seroconversions observed were not due to PEP failure but other high-risk exposures to HIV [36]. As PEP users are at greater risk of subsequent HIV infection given their propensity of continued at-risk behavior, patients should consider pre-exposure prophylaxis (PrEP) as an alternative prevention strategy. Daily PrEP use would be particularly beneficial for a third of patients in this study with repeat PEP episodes. A combination of risk reducing behavioral interventions and prophylaxis may therefore be more beneficial to patients than PEP alone [27, 37].

This study had several limitations. Adherence and re-exposure were measured by self-report, thereby being potentially susceptible to social desirability bias as patients may not have been forthcoming to their physicians about their behavioral practices and treatment compliance. Adherence data was missing for several patients. Though intent-to-treat analysis was used to compensate for the missing data, we may have underestimated the proportion of patients adhering to treatment. Some patients inconsistently attended the scheduled follow-up visits for HIV testing, limiting our ability to accurately determine the time of seroconversion. We were also unable to adjust for other factors which may have influenced treatment indication and regimen selection.

## Conclusions

PEP regimen was significantly associated with adherence to treatment. Patients were more likely to be adherent to TVD-based regimens which are known to have better tolerability than CBV-based regimens. Ten patients seroconverted during the follow-up period after taking PEP; however, only a single case of PEP failure was observed as the remaining cases did not reduce at-risk behaviors and increased possible re-exposure to HIV. PEP is therefore a successful method to prevent HIV infection after sexual exposure. This study also underlines the impact of a global approach, including a close and multidisciplinary follow-up, as a key component of the PEP protocol.
